# Clinical Value of Multiomics-Based Biomarker Signatures in Inflammatory Bowel Diseases: Challenges and Opportunities

**DOI:** 10.14309/ctg.0000000000000579

**Published:** 2023-03-07

**Authors:** Arno R. Bourgonje, Harry van Goor, Klaas Nico Faber, Gerard Dijkstra

**Affiliations:** 1 Department of Gastroenterology and Hepatology, University of Groningen, University Medical Center Groningen, Groningen, the Netherlands;; 2 Department of Pathology and Medical Biology, University of Groningen, University Medical Center Groningen, Groningen, the Netherlands.

**Keywords:** inflammatory bowel diseases, multiomics, biomarkers, systems biology, personalized medicine, validation

## Abstract

Inflammatory bowel diseases (IBDs), encompassing Crohn's disease and ulcerative colitis, are complex and heterogeneous diseases characterized by a multifactorial etiology, therefore demanding a multimodal approach to disentangle the main pathophysiological components driving disease onset and progression. Adoption of a systems biology approach is increasingly advocated with the advent of multiomics profiling technologies, aiming to improve disease classification, to identify disease biomarkers, and to accelerate drug discovery for patients with IBD. However, clinical translation of multiomics-derived biomarker signatures is lagging behind because there are several obstacles that need to be addressed to realize clinically useful signatures. Multiomics integration and IBD-specific identification of molecular networks, standardization and clearly defined outcomes, strategies to tackle cohort heterogeneity, and external validation of multiomics-based signatures are critical aspects. While striving for personalized medicine in IBD, careful consideration of these aspects is, however, needed to adequately match biomarker targets (e.g., the gut microbiome, immunity, or oxidative stress) with their corresponding utilities (e.g., early disease detection and endoscopic and clinical outcome). Theory-driven disease classifications and predictions are still governing clinical practice, while this could be improved by adopting an unbiased, data-driven approach relying on molecular data structures integrated with patient and disease characteristics. In the foreseeable future, the main challenge will lie in the complexity and impracticality of implementing multiomics-based signatures into clinical practice. Still, this could be achieved by developing easy-to-use, robust, and cost-effective tools incorporating omics-derived predictive signatures and through the design and execution of prospective, longitudinal, biomarker-stratified clinical trials.

## INTRODUCTION

Inflammatory bowel diseases (IBDs), comprising Crohn's disease (CD) and ulcerative colitis, are chronic immune-mediated diseases of the gastrointestinal tract, characterized by a broad clinical heterogeneity and a high degree of pathophysiological complexity (1). CD is characterized by transmural ulcerative inflammation that can occur in any part of the gastrointestinal tract, whereas ulcerative colitis is marked by rather superficial inflammation that is limited to the colon. Although the exact etiology of IBD remains elusive, an interplay between genetic background, gut microbiota, immunity, and environmental factors (e.g., lifestyle and diet) is considered to underlie its pathogenesis (2,3). The peak age of onset lies within the second to fourth decade of life, and the disease course typically alternates between episodes of quiescent and active disease, which are difficult to predict and to adequately treat. The complex, unpredictable, and heterogeneous nature of IBD complicates early detection of the diagnosis, monitoring of disease activity and complications, and prediction of disease course and treatment response. This highlights the urgent need for biomarkers: objectively measured indicators of (ab)normal biological processes or systems or pharmacologic responses to therapeutic interventions (4,5).

In the context of IBD, biomarkers are already used for a variety of clinical purposes and can be derived from several determinants of IBD susceptibility, for example, the host genome, transcriptome, proteome, immune system, or gut microbiome, or from pathogenic mechanisms such as inflammation, oxidative stress, and fibrosis (6). Given the complex pathobiology of IBD, insights from multiple layers of biological data, referred to as a systems biology approach, are required to unravel disease pathogenesis (7,8). In addition, the therapeutic armamentarium of IBD covers drugs targeting many different molecular pathways, whereas we lack the knowledge to make educated decisions about which drug is best for each individual patient. Systems biology consists of holistic and mathematical modeling of complex biological systems (9,10). Recent technological (e.g., next-generation sequencing and high-density protein arrays) and computational (e.g., machine learning and artificial intelligence) advances have facilitated the integration of big data, enabling the establishment of molecular constructs that are specific to IBD (11,12). However, the investigation of such big complex molecular data entities should be accompanied by careful integration of patient phenotypes, clearly defined outcomes, and independent validation to achieve clinically translatable, omics-based biomarker signatures.

In this review, we highlight the potential of multiomics profiling for biomarker discovery in IBD, followed by an outline of key challenges and unmet needs warranting attention in this context. Finally, we outline some key examples of recent developments in clinical implementation of multiomics-based biomarker signatures in IBD. These opportunities to improve disease prediction using multiomics data may eventually translate into improved outcomes for patients with IBD.

## THE PROMISE OF MULTIOMICS-BASED BIOMARKER SIGNATURES IN IBD

**Figure 1.**
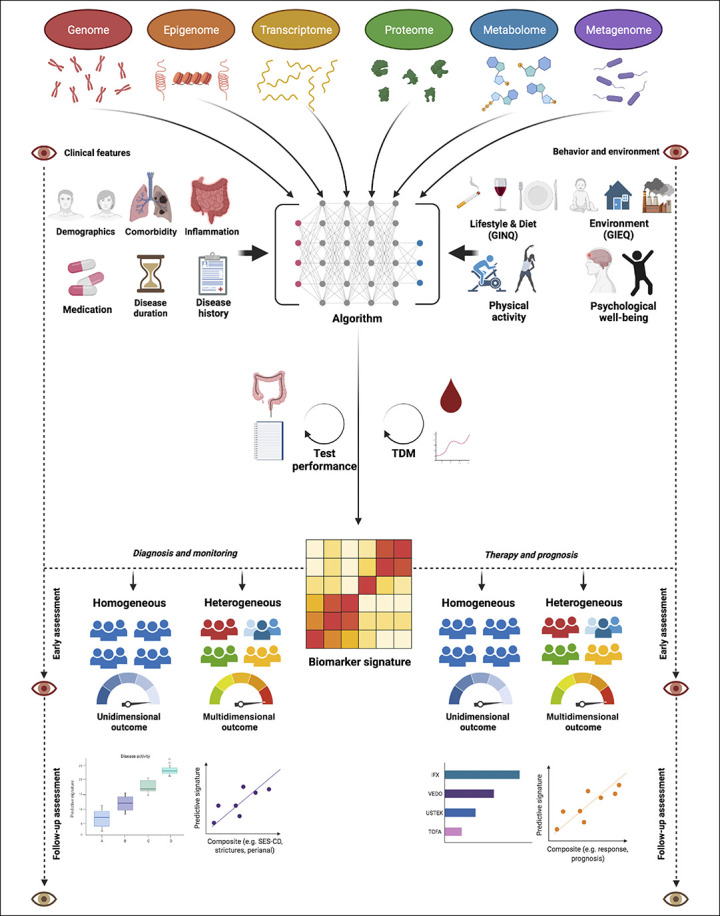
Multi-omics-driven biomarker discovery as strategy to develop personalized medicine for patients with IBD. An unbiased, data-driven approach may reveal key pathophysiological signatures while necessitating the integration of “*phenome*” data (i.e. clinical [upper left] and environmental [upper right] features) in order to improve clinical outcomes of patients with IBD. Artificial intelligence algorithms may help to allow the generation of individualized biomarker signatures from this molecular data-driven approach. After successful integration and validation, such individualized predictive biomarker signatures may aid in diagnosis and monitoring [lower left] as well as predicting therapeutic success and disease prognosis [lower right]. Depending on the disease outcome, different approaches could be followed to clinically integrate these signatures. One approach entails studies of patients with IBD having heterogeneous features that may be suitable for multi-dimensional (disease modification) outcome assessment, e.g. composite outcomes of disease activity or therapeutic response. Another approach consists of studies focusing on patients with IBD having homogeneous features to allow the assessment of the true objectivity of a certain multi-omics-derived biomarker by matching a specific group of patients to its most suitable outcome measure. Both approaches, however, require ‛quality control': for example, in case of diagnostic signatures, it is crucial that the gold standard outcome is objectively and accurately determined, whereas in case of therapeutic signatures, it is crucial that drug exposure is sufficiently guaranteed (e.g. by TDM). Eventually, developed signatures need long-term follow-up to assess the durability of their effectiveness in predicting disease outcomes. Abbreviations: GIEQ, Groningen IBD Environmental Questionnaire (24); GINQ, Groningen IBD Nutritional Questionnaire (25); TDM, therapeutic drug monitoring.

The complex, heterogeneous, and multifactorial nature of IBD rationalizes a systems biology approach for its management. The advent of multiomics profiling technologies such as genomics (whole-genome genotyping using ImmunoChip or Global Screening Array sequencing, for example, and whole-exome sequencing), transcriptomics (e.g., bulk RNA sequencing), proteomics (e.g., proximity extension assay technology, modified-aptamer binding technology, or mass spectrometry–based techniques), or metagenomics (metagenomic shotgun sequencing, among others) allows for a better understanding of IBD pathophysiology. An increasing number of multiomics studies revealed signatures predictive of disease phenotypes, disease course, therapeutic success, and prognosis in patients with IBD (11,13–22). Despite the experimental and computational advances made in this field, the exact clinical utility of multiomics-derived signatures, however, remains poorly characterized, mainly due to a lack of their integration, sparsity, and international synchronization. Leveraging machine learning–based methods and bioinformatic tools, multiomics profiles carry potential to improve disease classification and prediction by interrogating the enormous pile of biological data arising from them. Unlike traditionally used theory- or symptom-based approaches for disease classification (e.g., the Montreal classification and Rutgeerts score), molecular data–driven biomarker discovery may reveal the key pathophysiological components of IBD, using molecular data structures while relying on detailed phenotypes (Figure 1).

An unbiased generation of composite biomarker signatures may confer predictive accuracy in relation to disease activity, complications, or therapeutic response, enabling delivery of the most effective treatment to every patient with IBD (23). In addition, multiomics characterization could inspire functional studies to acquire mechanistic insight into the biological relevance of the identified signatures and thereby increase their potential utility (8). Clinical translation of multiomics-derived signatures could guide physicians in making treatment decisions for their patients, based on estimated individual therapeutic efficacy, following the concept of personalized medicine. Yet, this approach has not translated into robust clinical implementations because there are several unresolved issues impeding clinical integration. A key issue pertains to interindividual patient variation that keeps explaining the majority of data variation in multiomics studies, risking that potentially important observations are masked within subject-to-subject differences (12). Many variables may affect the dynamics of multiomics configurations, requiring prospective longitudinal studies and accurate recordkeeping during the disease course of patients to comprehensively model interactions between biological features, host characteristics, and clinical outcomes. Furthermore, stratification for specific clinical parameters alongside validation of identified signatures in independent cohorts is eventually needed to pinpoint to functionally relevant markers of IBD.

## CHALLENGES AND PITFALLS COMPLICATING CLINICAL TRANSLATION OF MULTIOMICS-BASED BIOMARKER SIGNATURES

Despite the advances made in multiomics-driven biomarker discovery for IBD, there are several obstacles that need to be addressed to realize clinical translation of multiomics-based signatures. Key aspects in this context include the need for multiomics data integration and network analysis, the identification of appropriate bioinformatic approaches phenotypic patient stratification, standards and definitions for relevant disease modification outcomes, and external validation of predictive signatures.

### Integrative multiomics and molecular networks specific to IBD

Single-omics characterization of IBD is well established, and this has provided insights into the functional dysregulation and distinct alterations in the genome, gut microbiome, transcriptome, proteome, and metabolome, among others, in patients compared with non-IBD controls. Although valuable knowledge has been gained in these studies, integration of multiomics layers would give more comprehensive insight into the complexity and key nodes of interactions. However, there are only few studies available that integrated at least 2 different omics data sets of patients with IBD, as was recently reviewed elsewhere (7). A key example includes the Integrative Human Microbiome Project, in which 132 patients were followed for over a year and longitudinally sampled for metagenomics, metatranscriptomics, metaproteomics, and metabolomics profiling (11). Its unique study design allowed to identify the dynamic changes in these complementary molecular profiles, which proved to be of much greater magnitude than were cross-sectional differences among the studied phenotypes. Although many distinct features were identified, and some demonstrated temporal stability, independent validation was lacking. Another aspect relevant to integration of multiomics data includes the establishment of IBD-specific molecular network information. Although many types of interactions (e.g., microbe-metabolite, gene-protein, and microRNA–messenger RNA) would provide pathophysiological insights, they are often not context specific, but rather generic, usually as a result of experimental conditions. Therefore, more targeted approaches are required to profile IBD-specific interactions leveraging appropriate bioinformatic tools (7).

### Bioinformatic challenges associated with integrative multiomics

One of the key challenges associated with performing integrative multiomics studies pertains to the identification of tailored bioinformatics approaches to tackle complex data structures such as multiomics datasets. One of the key challenges in this regard is the aforementioned interpatient variation that keeps to explain to majority of multiomics data variation, which reflect the problem that a wide variety of variables affect the dynamics of multiomics data configurations (12). On the other hand, missing data and the requirement for bioinformatic data imputation decrease the accuracy of the acquired data and thereby may introduce bias (10). There are also more statistical obstacles, such as insufficient power, class imbalances, or data sparsity, that may complicate or exclude the use of certain bioinformatic approaches and/or affect interpretation of integrative analyses. In addition, there may be a lack of biological granularity in that bulk sequencing of DNA and RNA extracted from biopsies or blood is performed in many studies, whereas cell type specificity driving the pathophysiology of IBD becomes increasingly apparent. Importantly, there are also more practical bioinformatic challenges such as the availability and practicality of data storage and data analysis workflows alongside the growing need for more volume and computational power, respectively. In this respect, the implementation of more user-friendly software platforms, for example, to facilitate clinical IBD researchers in handling complex multiomics data sets would be important to better translate these efforts into clinical practice. Finally, there is a lack of consensus regarding which computational methodologies are preferred over others in dealing with specific high-throughput multiomics datasets. Closer collaboration between experienced bioinformaticians and clinical IBD researchers is crucial to facilitate performance of multiomics integration studies and establish consensus-based recommendations or even guidelines for multiomics data analysis.

### Phenotypic patient stratification to account for key clinical confounders

**Figure 2.**
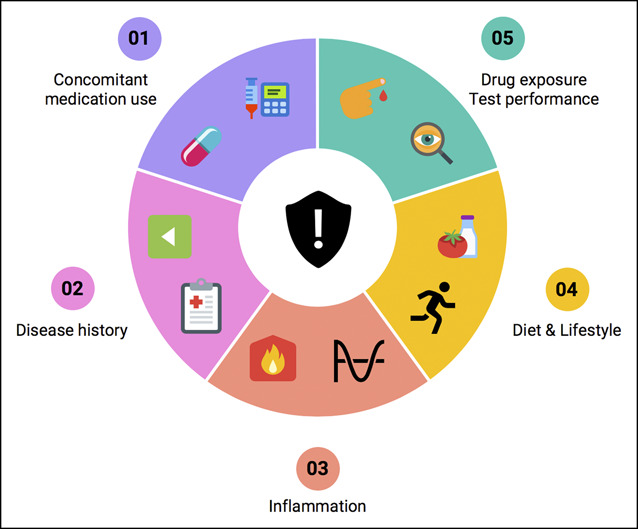
Key clinical confounders jeopardizing observations in multi-omics-driven biomarker studies. Important albeit non-exhaustive clinical factors warranting consideration in multi-omics studies include (1) concomitant medication use, which may induce molecular changes influencing the biological data under investigation; (2) disease history, including previous drug exposure but also disease duration and surgical history; (3) baseline inflammatory status, which may impact biological features and the outcome(s) under investigation; (4) diet, lifestyle, and environmental exposures, which may have an underrecognized influence on disease pathobiology; (5) drug exposure and test performance, i.e. is the drug under investigation biologically available (pharmacodynamic and pharmacokinetic properties) or in case of diagnostics, is the diagnostic method under investigation (e.g. endoscopy) appropriately performed and quantified to allow accurate investigation? Clinical disease heterogeneity of IBD poses a major challenge to multi-omics-driven biomarker discovery, necessitating stratification and the use of large, well-characterized patient and control cohorts.

An important challenge pertaining to integrative omics analyses constitutes interpatient variation that contributes to the majority of data variation, which may jeopardize observations of potentially important pathophysiological features that may otherwise remain unidentified. Although the list of potential clinical confounding variables is long, and many remain context dependent, some key factors deserve mentioning in the context of clinical multiomics integration in IBD (Figure 2).

First, an important but often overlooked aspect is the use of concomitant medication. A striking example includes corticosteroids, which may confound biomarker discovery mainly because of their inherently strong anti-inflammatory effects, which may affect disease activity and therapeutic response (26). Although it remains difficult to pinpoint the exact effects on certain molecules, medication usage may result in inaccurate biomarker evaluation. Similarly, other elements of clinical history may be critical, for example, disease duration, which is generally inversely associated with therapeutic success rates (27). Moreover, medication history impacts the plausibility of responding to novel treatments, that is, previous use of certain medications such as tumor necrosis factor alpha (TNFα) antagonists decreases the chance of responding to a subsequent therapy (28). This phenomenon is illustrated by studies that investigated the molecular changes on vedolizumab and ustekinumab treatments, which generally exert higher anti-inflammatory effects, that is, showing better results, in patients who are naive to TNFα antagonists (29,30). As such, not only concomitant medication usage but also previous drug exposure and disease history are important factors to account for in biomarker studies. Third, the degree of intestinal inflammation, which relentlessly fluctuates in patients, impacts physiological processes such as gene expression, protein production, and paracrine communication and triggers structural (tissue) changes. As a consequence, querying multiomics data for biomarkers may become problematic because the biological constituents under investigation (i.e., gene expressions, proteins, or metabolites) may change on inflammation and conceal the true objectivity of a particular signature (i.e., being related to the pathophysiology of the disease or to a pharmacological mechanism). Considering this, any particular multiomics signature should ideally outperform classical parameters of inflammation such as C-reactive protein or fecal calprotectin, which have repeatedly been associated with relevant outcomes such as therapeutic response (31,32). This emphasizes the need for investigating multiomics signatures in both inflamed and noninflamed situations to test this potential inflammatory dependency. Fourth, the environmental contingency of a study population should be carefully considered. Differences in habitual diet and environmental exposures may have profound effects on disease pathobiology and may thus impact on a specific layer of biological data under evaluation (3). Finally, drug exposure is important because insufficient exposure could lead to falsified conclusions about the performance of multiomics-derived predictive signatures. For instance, in studies searching for biomarkers of therapeutic response, it would be important to confirm that patients achieve roughly equal levels of drug exposure because some might otherwise be regarded as nonresponders due to pharmacodynamic failure. The implementation of therapeutic drug monitoring is crucial to address this issue by excluding the possibility of insufficient drug exposure as reason for therapeutic failure.

### Setting standards and definitions for clinical outcomes to enhance effectivity of multiomics-based biomarker signatures

Various clinical outcomes have been used in multiomics-driven biomarker evaluation, for example, disease activity, therapeutic response, or fibrotic disease complications (11,14,15,17). These, in turn, result in a variety of study end points, for example, the degree of endoscopic disease activity, the height of clinical scores defining response to therapy, or intestinal thickness as indicator of fibrosis. However, this heterogeneity in outcome assessments does not fully appreciate the pathophysiological complexity and clinical heterogeneity of IBD or facilitate the prevailing aim of modifying disease course and changing prognosis for patients with IBD (33).

At single-center level, there is often already a lack of synchronization of study outcome definitions in clinically oriented multiomics studies, which may have practical reasons. For example, standardized endoscopic evaluation of disease activity after induction therapy with biologics varies across hospitals: although in some this is never practiced, in others, it is performed at prespecified time intervals during therapy irrespective of the patient's clinical status. Moreover, disagreement may arise in scoring of clinical disease activity (e.g., using Crohn's Disease Activity Index, Harvey-Bradshaw Index, or Simple Clinical Colitis Activity Index scores) or with regard to criteria that should be adopted for defining clinical response to therapy. Such scenarios may result in severe under- or overestimation of the real value of multiomics-based biomarker signatures and subsequently complicate their validation.

At the multicenter level, the heterogeneity in outcome definitions of multiomics-driven biomarker studies further expands. In this respect, defining endoscopic disease activity is a striking example. Although some centers use prespecified cutoffs of specific endoscopic scores to define endoscopic remission, others take the absence of any ulcerations (mucosal healing) as sole criterion (34). Although mucosal healing is increasingly recognized as an important therapeutic end point in clinical trials—because it strongly associates with sustained remission and resection-free survival (35–37)—there is not yet full consensus on using it as definite endoscopic outcome because the evidence for efficacious induction of mucosal healing varies by type of treatment while interpatient variation complicates efforts to achieve it (38). To overcome this lack of uniformity, endoscopic assessments are increasingly subject to central reading, that is, independent, off-site, blinded review of imaging end points in clinical trials (39).

From a statistical perspective, there is also much heterogeneity in relating clinically relevant outcomes to multiomics-based signatures. For example, dichotomizing outcomes for statistical convenience may lead to a potential loss of information by removing fine-grained, intracategory information that may more accurately represent true biological scales (40). Machine learning–driven models often used in multiomics studies may suffer from these efforts because it may result in underestimation of the true predictive value of certain signatures (41). Studies are warranted to investigate the noninferiority of using either continuous or categorical definitions for each unique outcome, including studies focusing on the minimal differences in outcome that would be considered clinically (or biologically) relevant.

To achieve more international consensus on the definition of disease modification outcome measures, several collaborative efforts (e.g., the Selecting End PoInts foR Disease-ModIfication Trials and Selecting Therapeutic Targets in Inflammatory Bowel Disease initiatives (33,42,43)) are aimed at establishing objective, holistic, multidimensional outcome assessment by following a patient-centered approach that would be more compatible with IBD.

### External validation of multiomics-based biomarker signatures

To date, few multiomics data integration studies in IBD performed independent, external validation of their key findings (although there are exceptions (13)). Instead, cross-validation procedures (i.e., train- and test splits from the same cohort) are often performed when an independent replication cohort is lacking. However, before any multiomics-based biomarker signature could be suitable for clinical implementation, it is important to validate its utility in diverse populations, for example, ethnic and geographically distinct cohorts including patients with differing genetic background and environmental exposures. Because it is likely that such factors will at least partially determine the behavior of identified signatures (because they influence human biology), external validation is required to test their generalizability and reproducibility. Ideally, this should be performed in large, well-characterized cohorts of patients. Currently, there are large ongoing efforts attempting to realize this multiomics testing on a global level, for example, the IBD Plexus or the COLLIBRI and 3TR consortia. This may bridge the gap between the development of multiomics-driven signatures and their clinical implementation, while also avoiding waste of extensive research efforts in finding potential biomarkers.

**Figure 3.**
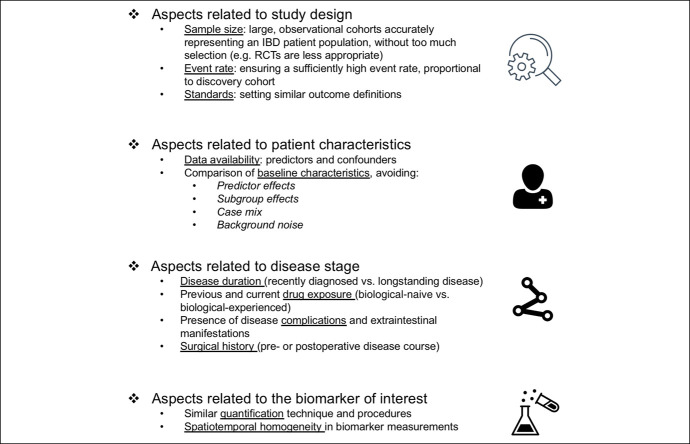
Proposed criteria to consider when performing external validation of predictive signatures derived from multi-omics studies. Central criteria constitute aspects related to study design, including the sample size and sample characteristics (avoiding selection bias), but also the (proportionality of) event rate and definition of outcome parameters. Subsequently, patient and disease characteristics need to be carefully compared with those of the discovery cohort, while having the necessary predictors and confounding variables at hand in both cohorts. This should be performed to avoid predictor and subgroup effects, “case mix”, and background noise in study populations. Finally, it is critical to perform multi-omics data generation or quantification of the biomarker of interest in a similar manner, avoiding large differences in measurement circumstances or protocols.

It is important to develop criteria for appropriate external validation studies (Figure 3). For example, the fraction of patients undergoing or achieving the event (e.g., response to treatment) and its proportionality to the discovery cohort should be carefully considered to avoid model overfitting and to allow the adjustment for confounding variables (44,45). This is crucial as it may impact model performance, and the baseline risk of the event may differ across populations. In addition, profiling of multiomics-based signatures should ideally be performed under similar conditions to avoid interference with predictive performances (46). Furthermore, the assessment of baseline patient and disease characteristics between discovery and external validation cohorts is important to avoid large differences in case mix, referring to the distribution of predictor values that may influence the predictive performance of the signature under investigation (47,48). This bias could be addressed by comparing baseline characteristics between discovery and validation cohorts to determine the degree of cohort similarity and generalizability. In general, the more homogeneous and similar both cohorts are, the higher the likelihood of successful validation will become. Striving for this cohort homogeneity is especially important for validation of rather dynamic biomarkers (e.g., proteomic- or transcriptomic-based biomarkers) to reduce the risk of confounding. Statistical frameworks have been developed to allow comparisons of baseline characteristics for external validation studies by calculating (dis)similarity metrics between cohorts (49). In addition, bias risk estimation tools have been developed that may help to determine whether a particular prediction model is suitable for external validation (e.g., the Prediction model Risk Of Bias ASsessment Tool [PROBAST] (50)).

## TOWARDS CLINICAL IMPLEMENTATION OF MULTIOMICS-DERIVED BIOMARKER SIGNATURES

The main challenge associated with multiomics-driven biomarker discovery lies in the complexity and impracticality of such data-driven approaches in clinical practice. Clinical integration of multiomics profiles entails financial, legal, ethical, and other logistic constraints, without even considering potential strategies (51,52). Thus, the development of easily applicable, robust, and cost-effective clinical implementations incorporating multiomics-derived biomarker signatures should be prioritized. Several examples exist that illustrate the potential of multiomics data integration to improve disease classification, to predict disease prognosis, and to individualize treatment. For example, a recent study established a pharmacogenetic passport integrating individual genetic variants to predict the risk of adverse drug responses such as thiopurine-induced myelosuppression, pancreatitis, and immunogenicity of TNFα antagonists (53). Such pharmacogenetic tools may aid in providing personalized treatment recommendations by optimizing drug selection and minimizing drug toxicity, resulting in a potential reduction of therapeutic failure and costs. Another example constitutes the development of a transcriptomics-based blood-derived 17-gene prognostic biomarker that could predict prognosis in newly diagnosed patients with IBD (54). This CD8^+^ T-cell gene expression signature accurately identified patients who experienced an aggressive disease course characterized by earlier disease recurrence and a higher frequency of disease flares (55). Importantly, this signature was subsequently replicated in multiple prospective, independent replication cohorts from the UK, and this has culminated into the initiation of the first-ever biomarker-stratified clinical trial in IBD (56). This trial will evaluate whether this transcriptional signature is indeed capable of improving clinical outcomes by facilitating personalized medicine for patients with CD. Insofar, most of the currently available omics studies are single-omics studies, whereas multiomics integration studies hold promise to find more accurate biomarkers. The potential value of clinical multiomics integration became particularly evident from a recent study integrating serum metabolomics, proteomics, and fecal metagenomics data of patients with IBD (57). Two distinct microbial signatures were identified that were able to characterize a subset of patients who would benefit more from anticytokine therapy compared with anti-integrin therapy. The addition of multiomics information to their prediction models resulted in an astonishing increase in predictive accuracy: although a baseline model solely containing clinical information and serum inflammatory markers reached an area under the curve of 0.62, the inclusion of multiomics profiles dramatically increased this to 0.96 for predicting therapeutic response. Although external validation is required before application in clinical practice could ensue, these examples illustrate that the field of multiomics-driven biomarker discovery is rapidly advancing and may particularly improve responses to inflammation-targeted therapies in IBD (58). The promise of multiomics integration and validation is supported by successful efforts like those reported in the field of oncology. For instance, an integrated microRNA–messenger RNA global profiling approach has previously been leveraged to identify microRNAs that were independently associated with prognosis in patients with breast cancer (59). Other examples include the WINTHER and SPRING trials that tested the use of integrative multiomics-based biomarker signatures to improve treatment strategies for patients with (non–small-cell lung) cancer (60,61). Therefore, also in the IBD field, it is important to continue searching for biomarkers because this will help health care providers to make accurate therapeutic decisions already at first disease presentation (e.g., deciding on type of biological therapy), further decreasing the rates of therapeutic nonresponse.

## CONCLUDING REMARKS

Unraveling the pathophysiological complexity of a heterogeneous disease like IBD necessitates a systems biology approach, which could be implemented using extensive and integrative multiomics characterization at different levels of biological organization. Multiomics-based biomarker signatures not only carry potential to advance our understanding of disease pathophysiology and accelerate drug discovery, shifting our focus to clinical integration of these signatures by developing clinical implementations could eventually improve disease outcomes for patients with IBD. Characteristics of multiomics signatures may also inspire the scientific community to develop new theories about the pathways associated with disease activity, therapeutic response, or any other clinical utility. A subsequent translation of these conceptualizing efforts may promote the design of functional studies that could help to gain mechanistic insight into the biological relevance of each signature and fuel its evidence-based grounds. For example, organoids or advanced gut-on-a-chip models could be used to validate transcriptional signatures, and gnotobiotic mice could be used to validate functional effects of gut microbial signatures. Identification of the causal mechanisms behind multiomics-derived signatures is important because experimental validation is usually lacking from initial discovery studies. Similarly, future studies are needed to understand the long-term durability of multiomics signatures, that is, the extent to which those signatures maintain their predictive value over time. Here, we attempted to provide a concise report of the challenges and opportunities originating from the application of multiomics profiling technologies in IBD, with special emphasis on the need for integration and network analysis, bioinformatic challenges, consideration of key clinical confounders, setting standards and definitions of clinically relevant outcomes, and the need for external validation of multiomics signatures. Molecular data-driven clinical implementations or clinical omics integration hold great potential by unraveling IBD pathobiology and addressing unmet clinical needs. Securing appropriate study designs, the distance between the clinic and laboratory (e.g., efficient transport of biomaterials to the site of analysis), data and the underlying infrastructure, financial and bioinformatic resources, and while using standardized outcomes, methodologies and technologies, this strategy could become reality and anchored in IBD care.

## CONFLICTS OF INTEREST

**Guarantor of the article:** Gerard Dijkstra, MD, PhD.

**Specific author contributions:** All authors were involved in conceptualization. A.R.B. reviewed the relevant literature and wrote the first draft of the manuscript. All authors contributed to manuscript revision, read, and approved the final version of the manuscript to be submitted for publication.

**Financial support:** None to report.

**Potential competing interests:** G.D. received research grants from Royal DSM and speaker fees from Janssen Pharmaceuticals, Takeda, Pfizer, and AbbVie. All other authors have no conflicts of interest to declare.

## References

[1] Chang JT. Pathophysiology of inflammatory bowel diseases. N Engl J Med 2020;383(27):2652–64. 33382932 10.1056/NEJMra2002697

[2] de Souza HSP Fiocchi C Iliopoulos D. The IBD interactome: An integrated view of aetiology, pathogenesis and therapy. Nat Rev Gastroenterol Hepatol 2017;14(12):739–49. 28831186 10.1038/nrgastro.2017.110

[3] Ananthakrishnan AN Bernstein CN Iliopoulos D . Environmental triggers in IBD: A review of progress and evidence. Nat Rev Gastroenterol Hepatol 2018;15(1):39–49. 29018271 10.1038/nrgastro.2017.136

[4] Bennike T Birkelund S Stensballe A . Biomarkers in inflammatory bowel diseases: Current status and proteomics identification strategies. World J Gastroenterol 2014;20(12):3231–44. 24696607 10.3748/wjg.v20.i12.3231 PMC3964395

[5] Biomarkers Definitions Working Group. Biomarkers and surrogate endpoints: Preferred definitions and conceptual framework. Clin Pharmacol Ther 2001;69(3):89–95. 11240971 10.1067/mcp.2001.113989

[6] Maaser C Sturm A Vavricka SR . ECCO-ESGAR Guideline for Diagnostic Assessment in IBD Part 1: Initial diagnosis, monitoring of known IBD, detection of complications. J Crohns Colitis 2019;13(2):144–64. 30137275 10.1093/ecco-jcc/jjy113

[7] Sudhakar P Alsoud D Wellens J . Tailoring multi-omics to inflammatory bowel diseases: All for one and one for all. J Crohns Colitis 2022;16(8):1306–20. 35150242 10.1093/ecco-jcc/jjac027 PMC9426669

[8] Fiocchi C Iliopoulos D. IBD systems biology is here to stay. Inflamm Bowel Dis 2021;27(6):760–70. 33438720 10.1093/ibd/izaa343

[9] Ideker T Galitski T Hood L. A new approach to decoding life: Systems biology. Annu Rev Genomics Hum Genet 2001;2(1):343–72. 11701654 10.1146/annurev.genom.2.1.343

[10] Seyed Tabib NS Madgwick M Sudhakar P . Big data in IBD: Big progress for clinical practice. Gut 2020;69(8):1520–32. 32111636 10.1136/gutjnl-2019-320065 PMC7398484

[11] Lloyd-Price J Arze C Ananthakrishnan AN . Multi-omics of the gut microbial ecosystem in inflammatory bowel disease. Nature 2019;569(7758):655–62. 31142855 10.1038/s41586-019-1237-9 PMC6650278

[12] Metwaly A Haller D. Multi-omics in IBD biomarker discovery: The missing links. Nat Rev Gastroenterol Hepatol 2019;16(10):587–8. 31312043 10.1038/s41575-019-0188-9

[13] Franzosa EA Sirota-Madi A Avila-Pacheco J . Gut microbiome structure and metabolic activity in inflammatory bowel disease. Nat Microbiol 2019;4(2):293–305. 30531976 10.1038/s41564-018-0306-4 PMC6342642

[14] Borren NZ Plichta D Joshi AD . Multi-“-omics” profiling in patients with quiescent inflammatory bowel disease identifies biomarkers predicting relapse. Inflamm Bowel Dis 2020;26(10):1524–32. 32766830 10.1093/ibd/izaa183 PMC7500522

[15] Sudhakar P Verstockt B Cremer J . Understanding the molecular drivers of disease heterogeneity in Crohn's disease using multi-omic data integration and network analysis. Inflamm Bowel Dis 2021;27(6):870–86. 33313682 10.1093/ibd/izaa281 PMC8128416

[16] Metwaly A Dunkel A Waldschmitt N . Integrated microbiota and metabolite profiles link Crohn's disease to sulfur metabolism. Nat Commun 2020;11(1):4322. 32859898 10.1038/s41467-020-17956-1 PMC7456324

[17] Douglas GM Hansen R Jones CMA . Multi-omics differentially classify disease state and treatment outcome in pediatric Crohn's disease. Microbiome 2018;6(1):13. 29335008 10.1186/s40168-018-0398-3 PMC5769311

[18] Jin L Li L Hu C . Integrative analysis of transcriptomic and proteomic profiling in inflammatory bowel disease colon biopsies. Inflamm Bowel Dis 2019;25(12):1906–18. 31173627 10.1093/ibd/izz111

[19] Bourgonje AR Hu S Spekhorst LM . The effect of phenotype and genotype on the plasma proteome in patients with inflammatory bowel disease. J Crohns Colitis 2022;16(3):414–29. 34491321 10.1093/ecco-jcc/jjab157 PMC8919819

[20] Yilmaz B Juillerat P Øyås O . Microbial network disturbances in relapsing refractory Crohn's disease. Nat Med 2019;25(2):323–36. 30664783 10.1038/s41591-018-0308-z

[21] Hu S Vich Vila A Gacesa R . Whole exome sequencing analyses reveal gene-microbiota interactions in the context of IBD. Gut 2021;70(2):285–96. 32651235 10.1136/gutjnl-2019-319706 PMC7815889

[22] Revilla L Mayorgas A Corraliza AM . Multi-omic modelling of inflammatory bowel disease with regularized canonical correlation analysis. PLoS One 2021;16(2):e0246367. 33556098 10.1371/journal.pone.0246367 PMC7870068

[23] Weersma RK Xavier RJ, IBD Multi Omics Consortium, Vermeire S . Multiomics analyses to deliver the most effective treatment to every patient with inflammatory bowel disease. Gastroenterology 2018;155(5):e1–4. 10.1053/j.gastro.2018.07.039 30077628

[24] van der Sloot KWJ Weersma RK Dijkstra G . Development and validation of a web-based questionnaire to identify environmental risk factors for inflammatory bowel disease: The Groningen IBD Environmental Questionnaire (GIEQ). J Gastroenterol 2019;54(3):238–48. 30109418 10.1007/s00535-018-1501-z PMC6394725

[25] Peters V Alizadeh BZ de Vries JHM . Nutritional assessment in inflammatory bowel disease (IBD): Development of the Groningen IBD Nutritional Questionnaire (GINQ). Nutrients 2019;11(11):2739. 31726688 10.3390/nu11112739 PMC6893781

[26] Alsoud D Vermeire S Verstockt B. Biomarker discovery for personalized therapy selection in inflammatory bowel diseases: Challenges and promises. Curr Res Pharmacol Drug Discov 2022;3:100089. 35146421 10.1016/j.crphar.2022.100089 PMC8818904

[27] Ungaro RC Aggarwal S Topaloglu O . Systematic review and meta-analysis: Efficacy and safety of early biologic treatment in adult and paediatric patients with Crohn's disease. Aliment Pharmacol Ther 2020;51(9):831–42. 32202328 10.1111/apt.15685 PMC7160034

[28] Rosario M French JL Dirks NL . Exposure-efficacy relationships for vedolizumab induction therapy in patients with ulcerative colitis or Crohn's disease. J Crohns Colitis 2017;11(8):921–9. 28333288 10.1093/ecco-jcc/jjx021

[29] Li K Friedman JR Chan D . Effects of ustekinumab on histologic disease activity in patients with Crohn's disease. Gastroenterology 2019;157(4):1019–31.e7. 31279870 10.1053/j.gastro.2019.06.037

[30] Monast C Li K Myshkin E . Molecular surrogates of histologic activity in Crohn's disease. United European Gastroenterol J 2017;5(5 Suppl 1):A523.

[31] Narula N Wong ECL Aruljothy A . Ileal and rectal ulcer size affects the ability to achieve endoscopic remission: A post hoc analysis of the SONIC trial. Am J Gastroenterol 2020;115(8):1236–45. 32759621 10.14309/ajg.0000000000000617

[32] Kopylov U Seidman E. Predicting durable response or resistance to antitumor necrosis factor therapy in inflammatory bowel disease. Therap Adv Gastroenterol 2016;9(4):513–26. 10.1177/1756283X16638833 PMC4913332 27366220

[33] Turner D Ricciuto A Lewis A . STRIDE-II: An update on the Selecting Therapeutic Targets in Inflammatory Bowel Disease (STRIDE) Initiative of the International Organization for the Study of IBD (IOIBD): Determining therapeutic goals for treat-to-target strategies in IBD. Gastroenterology 2021;160(5):1570–83. 33359090 10.1053/j.gastro.2020.12.031

[34] Dulai PS Levesque BG Feagan BG . Assessment of mucosal healing in inflammatory bowel disease: Review. Gastrointest Endosc 2015;82(2):246–55. 26005012 10.1016/j.gie.2015.03.1974 PMC6709676

[35] Karin M Clevers H. Reparative inflammation takes charge of tissue regeneration. Nature 2016;529(7586):307–15. 26791721 10.1038/nature17039 PMC5228603

[36] Pineton de Chambrun G Blanc P Peyrin-Biroulet L. Current evidence supporting mucosal healing and deep remission as important treatment goals for inflammatory bowel disease. Expert Rev Gastroenterol Hepatol 2016;10(8):915–27. 27043489 10.1586/17474124.2016.1174064

[37] Colombel JF D'Haens G Lee WJ . Outcomes and strategies to support a treat-to-target approach in inflammatory bowel disease: A systematic review. J Crohns Colitis 2020;14(2):254–66. 31403666 10.1093/ecco-jcc/jjz131 PMC7008150

[38] Villablanca EJ Selin K Hedin CRH. Mechanisms of mucosal healing: Treating inflammatory bowel disease without immunosuppression? Nat Rev Gastroenterol Hepatol 2022;19(8):493–507. 35440774 10.1038/s41575-022-00604-y

[39] Feagan BG Sandborn WJ D'Haens G . The role of centralized reading of endoscopy in a randomized controlled trial of mesalamine for ulcerative colitis. Gastroenterology 2013;145(1):149–57.e2. 23528626 10.1053/j.gastro.2013.03.025

[40] Fedorov V Mannino F Zhang R. Consequences of dichotomization. Pharm Stat 2009;8(1):50–61. 18389492 10.1002/pst.331

[41] Altman DG Royston P. The cost of dichotomising continuous variables. BMJ 2006;332(7549):1080. 16675816 10.1136/bmj.332.7549.1080 PMC1458573

[42] Le Berre C Peyrin-Biroulet L; SPIRIT-IOIBD study group. Selecting end points for disease-modification trials in inflammatory bowel disease: The SPIRIT consensus from the IOIBD. Gastroenterology 2021;160(5):1452–60.e21. 33421515 10.1053/j.gastro.2020.10.065

[43] Verstockt B Parkes M Lee J. How do we predict a patient's disease course and whether they will respond to specific treatments? Gastroenterology 2022;162(5):1383–95. 34995535 10.1053/j.gastro.2021.12.245

[44] Collins GS Ogundimu EO Altman DG. Sample size considerations for the external validation of a multivariable prognostic model: A resampling study. Stat Med 2016;35(2):214–26. 26553135 10.1002/sim.6787 PMC4738418

[45] Austin PC Steyerberg EW. Events per variable (EPV) and the relative performance of different strategies for estimating the out-of-sample validity of logistic regression models. Stat Methods Med Res 2017;26(2):796–808. 25411322 10.1177/0962280214558972 PMC5394463

[46] Luijken K Wynants L van Smeden M . Changing predictor measurement procedures affected the performance of prediction models in clinical examples. J Clin Epidemiol 2020;119:7–18. 31706963 10.1016/j.jclinepi.2019.11.001

[47] Ramspek CL Jager KJ Dekker FW . External validation of prognostic models: What, why, how, when and where? Clin Kidney J 2020;14(1):49–58. 33564405 10.1093/ckj/sfaa188 PMC7857818

[48] Riley RD Ensor J Snell KIE . External validation of clinical prediction models using big datasets from e-health records or IPD meta-analysis: Opportunities and challenges. BMJ 2016;353:i3140. 27334381 10.1136/bmj.i3140 PMC4916924

[49] Debray TPA Vergouwe Y Koffijberg H . A new framework to enhance the interpretation of external validation studies of clinical prediction models. J Clin Epidemiol 2015;68(3):279–89. 25179855 10.1016/j.jclinepi.2014.06.018

[50] Wolff RF Moons KGM Riley RD . PROBAST: A tool to assess the risk of bias and applicability of prediction model studies. Ann Intern Med 2019;170(1):51–8. 30596875 10.7326/M18-1376

[51] Verstockt B Noor NM Marigorta UM ; Scientific Workshop Steering Committee. Results of the seventh scientific workshop of ECCO: Precision medicine in IBD-disease outcome and response to therapy. J Crohns Colitis 2021;15(9):1431–42. 33730756 10.1093/ecco-jcc/jjab050 PMC8681673

[52] Torres J Halfvarson J Rodríguez-Lago I . Results of the seventh scientific workshop of ECCO: Precision medicine in IBD-prediction and prevention of inflammatory bowel disease. J Crohns Colitis 2021;15(9):1443–54. 33730755 10.1093/ecco-jcc/jjab048

[53] Bangma A Voskuil MD Uniken Venema WTC . Predicted efficacy of a pharmacogenetic passport for inflammatory bowel disease. Aliment Pharmacol Ther 2020;51(11):1105–15. 32363635 10.1111/apt.15762 PMC7318341

[54] Biasci D Lee JC Noor NM . A blood-based prognostic biomarker in IBD. Gut 2019;68(8):1386–95. 31030191 10.1136/gutjnl-2019-318343 PMC6691955

[55] Lee JC Lyons PA McKinney EF . Gene expression profiling of CD8+ T cells predicts prognosis in patients with Crohn disease and ulcerative colitis. J Clin Invest 2011;121(10):4170–9. 21946256 10.1172/JCI59255 PMC3196314

[56] Parkes M Noor NM Dowling F . PRedicting Outcomes For Crohn's dIsease using a moLecular biomarkEr (PROFILE): Protocol for a multicentre, randomised, biomarker-stratified trial. BMJ Open 2018;8(12):e026767. 10.1136/bmjopen-2018-026767 PMC6286485 30523133

[57] Lee JWJ Plichta D Hogstrom L . Multi-omics reveal microbial determinants impacting responses to biologic therapies in inflammatory bowel disease. Cell Host Microbe 2021;29(8):1294–304.e4. 34297922 10.1016/j.chom.2021.06.019 PMC8366279

[58] Mao R Chen M. Precision medicine in IBD: Genes, drugs, bugs and omics. Nat Rev Gastroenterol Hepatol 2021;19(2):81–2. 10.1038/s41575-021-00555-w 34785787

[59] Buffa FM Camps C Winchester L . microRNA-associated progression pathways and potential therapeutic targets identified by integrated mRNA and microRNA expression profiling in breast cancer. Cancer Res 2011;71(17):5635–45. 21737487 10.1158/0008-5472.CAN-11-0489

[60] Rodon J Soria JC Berger R . Genomic and transcriptomic profiling expands precision cancer medicine: The WINTHER trial. Nat Med 2019;25(5):751–8. 31011205 10.1038/s41591-019-0424-4 PMC6599610

[61] Solomon B Callejo A Berchem G . A WIN Consortium phase I study exploring avelumab, palbociclib, and axitinib in advanced non-small cell lung cancer. Cancer Med 2022;11(14):2790–800. 35307972 10.1002/cam4.4635 PMC9302335

